# Hepatic Oxi-Inflammation and Neophobia as Potential Liver–Brain Axis Targets for Alzheimer’s Disease and Aging, with Strong Sensitivity to Sex, Isolation, and Obesity

**DOI:** 10.3390/cells12111517

**Published:** 2023-05-30

**Authors:** Juan Fraile-Ramos, Anna Garrit, Josep Reig-Vilallonga, Lydia Giménez-Llort

**Affiliations:** 1 Institut de Neurociències, Universitat Autònoma de Barcelona, 08193 Barcelona, Spain; 2 Department of Psychiatry and Forensic Medicine, School of Medicine, Universitat Autònoma de Barcelona, 08193 Barcelona, Spain; 3 Department of Anatomy, School of Medicine, Universitat Autònoma de Barcelona, 08193 Barcelona, Spain

**Keywords:** Alzheimer’s disease, 3xTg-AD, liver–brain axis, obesity, HPA axis, corticosterone, oxidative stress, social isolation, amyloidosis, steatosis

## Abstract

Research on Alzheimer’s disease (AD) has classically focused on alterations that occur in the brain and their intra- and extracellular neuropathological hallmarks. However, the oxi-inflammation hypothesis of aging may also play a role in neuroimmunoendocrine dysregulation and the disease’s pathophysiology, where the liver emerges as a target organ due to its implication in regulating metabolism and supporting the immune system. In the present work, we demonstrate organ (hepatomegaly), tissue (histopathological amyloidosis), and cellular oxidative stress (decreased glutathione peroxidase and increased glutathione reductase enzymatic activities) and inflammation (increased IL-6 and TNF𝛼) as hallmarks of hepatic dysfunction in 16-month-old male and female 3xTg-AD mice at advanced stages of the disease, and as compared to age- and sex-matched non-transgenic (NTg) counterparts. Moreover, liver–brain axis alterations were found through behavioral (increased neophobia) and HPA axis correlations that were enhanced under forced isolation. In all cases, sex (male) and isolation (naturalistic and forced) were determinants of worse hepatomegaly, oxidative stress, and inflammation progression. In addition, obesity in old male NTg mice was translated into a worse steatosis grade. Further research is underway determine whether these alterations could correlate with a worse disease prognosis and to establish potential integrative system targets for AD research.

## 1. Introduction

Alzheimer’s disease (AD), the most prevalent form of dementia among older adults, is expected to increasingly affect the global population [1,2,3]. To date, research on AD has classically focused on alterations in the brain based on the original findings of the main pathological hallmarks of the disease: extracellular accumulation of β-amyloid (Aβ) plaques and intraneuronal neurofibrillary tangles of hyperphosphorylated tau protein [4,5]. New clinical evidence also suggests the influence of peripheral organs on the pathophysiological development of the disease, which can be analyzed using classical oxidative stress and inflammation theories of aging [6,7]. Biomarkers of oxidative stress identified in the urine of patients with AD and peripheral inflammation biomarkers that correlate with the risk of dementia suggest physiological effects not only in the brain but also in peripheral tissues [8,9]. In support of these emergent areas in AD research, animal models have confirmed crosstalk between central and peripheral inflammation [10,11,12].

The liver is considered a hotspot for oxidative stress because it regulates metabolism and inflammation by supporting the immune system [13]. Moreover, its role in the peripheral metabolism of Aβ has been described [14]. Oxidative stress and inflammation are risk factors for chronic hepatic diseases and negatively affect each other [15]. Recent findings have implicated this organ in the pathophysiology of AD, as an association between peripheral markers of liver function and central markers associated with AD has been described in people with the disease [16]. Moreover, liver dysfunction and impaired Aβ clearance have been suggested as early events in the pathophysiology of AD and are decisive in the progression of the disease [17,18]. Preliminary results from our laboratory for 3xTg-AD mice also suggested hepatic oxidative stress dysfunction at the onset of the disease [19,20]. Therefore, biochemical communication between these two organs via the so-called liver–brain axis has begun to attract attention.

In addition to this new perspective on the involvement of peripheral organs in AD, the classical cognitive conceptualization of this disease, centered on memory impairment at the clinical level, now embraces the disease’s broad and heterogeneous spectrum of neuropsychiatric and behavioral manifestations [21]. It has been well documented that mouse AD models have substantial defects in memory and cognition. In our extensive work with 3xTg-AD mice created by LaFerla’s laboratory [22], we described that this animal model for AD exhibits a conspicuous neuropsychiatric (NPS)-like phenotype [23,24]. However, it is not yet known how these cognitive changes are related to peripheral abnormalities. Among these, psychological stress and anxiety are commonly present in patients with AD, underlined by dysregulation of the hypothalamic–pituitary–adrenal (HPA) axis and consequent negative feedback of increased glucocorticoid levels in the limbic system that worsens AD progression [25]. Moreover, in mammals, glucocorticoids regulate the pro- and anti-inflammatory balance in the immune response. Our research has also demonstrated dysregulation of the HPA axis, neuroimmune communication derangement with increased peripheral oxidative stress, and inflammation, as observed in spleen and peritoneal cells [23,26,27,28,29]. This neuroimmune–endocrine effect has also been reproduced in other animal models of AD [30]. Studies in chickens have confirmed that chronic corticosterone administration leads to increased lipid deposition in the liver, which may be accompanied by oxidative stress and inflammation [31]. This reaffirms the involvement of peripheral organs in the disease and how they are influenced by neuropsychiatric and behavioral manifestations.

Extrinsic environmental factors are decisive in maintaining neuroimmune–endocrine homeostasis [26,29] and many physiological and psychological processes [32]. We have recently demonstrated short naturalistic isolation effects due to the phenotype of survivors [10,33,34]. In contrast, social isolation and poor responses to stress and anxiety, male sex, menopause, and obesity are models of premature aging in rodents and humans. In animals, premature aging can be monitored through behavioral tests and is characterized by peripheral immunosenescence, oxidative and inflammatory stress, and nervous system derangement [35]. Therefore, the extrinsic factors considered were standard (grouped), naturalistic (natural death of congeners), and forced (experimental) social isolation housing conditions.

Taking advantage of the conspicuous NPS-like phenotype of 3xTg-AD mice [24], in the present study, we investigated liver dysfunction at different levels of study, using cellular oxi-inflammation, histological analysis, and functional correlates with the HPA axis (corticosterone) and the liver–brain axis (NPS-like phenotype). Male and female 3xTg-AD mice were studied in a longitudinal design starting at 13 months and ending at 16 months, corresponding to advanced stages of the disease [36,37]. However, their NTg counterparts, the gold standard C57BL/6 mice, are prone to exhibit a sex-dependent obese profile at this age. The extrinsic factors considered were standard (grouped), naturalistic (natural death of congeners), and forced (experimental) social isolation housing conditions.

## 2. Materials and Methods

### 2.1. Animals

Genetically engineered homozygous triple-transgenic 3xTg-AD mice carrying the human PS1/M146V, APP_Swe_, and tau_P301L_ transgenes were produced at the University of California, Irvine [22]. Briefly, two independent transgenes encoding human APP_Swe_ and tau_P301L_ (both regulated by mouse Thy1.2) were co-injected into single-cell embryos from PS1KI (PS1M146V knock-in) mice.

A cohort of sixty-six male and female mice from Spanish colonies of C57BL/6 wild-type mice (from now referred to as NTg, non-transgenic mice) (*n* = 29) and homozygous 3xTg-AD mice (*n* = 37) from litters of a breeding program established at Universitat Autònoma de Barcelona after embryonic transfer to C57BL/6 strain background were used [38]. All animals were housed in groups of 3–4 individuals of the same sex and genotype until the experiment. The longitudinal design studied the animals from 13 to 16 months of age. They were maintained in cages (Makrolon, 35 × 15 × 15 cm^3^) under standard laboratory conditions (12 h light/dark, cycle starting at 8:00 a.m., food and water ad libitum, 22 ± 2 °C, 50–60% humidity). Biochemical and histopathological analyses were performed in a counterbalanced manner, blinded to the experiment.

All procedures were performed according to the Spanish legislation on the ”Protection of Animals Used for Experimental and Other Scientific Purposes” and the EU Council directive (2010/63/EU). The protocol CEEAH 3588/DMAH 9452 was approved on the 8th of March 2019 by Departament de Medi Ambient i Habitatge, Generalitat de Catalunya. ARRIVE guidelines developed by NC3Rs were followed, to reduce the number of animals used in the experimental procedures [39].

### 2.2. Extrinsic Factors: Naturalistic and Forced Social Isolation

Naturalistic isolation (nISO) resulting from increased mortality in 3xTg-AD mice [23] elicited a group of male 3xTg-AD mice that were naturally isolated for 2–3 months after the death of their cage mates. To mirror this scenario in both genotypes, a forced isolation (fISO) paradigm was conducted; at 13 months of age, a group of animals per each sex and genotype was individualized in cages and kept isolated for 3 months until they were euthanized. The rest of the animals remained in group-housed conditions. Namely, the following groups were established: Males NTg grouped (*n* = 6), Males NTg fISO (*n* = 8), Males 3xTg-AD grouped (*n* = 10), Males 3xTg-AD fISO (*n* = 10), Females NTg grouped (*n* = 7), Females NTg fISO (*n* = 8), Females 3xTg-AD grouped (*n* = 10), Females 3xTg-AD fISO (*n* = 7) and, additionally in Table 1, Males 3xTg-AD nISO (*n* = 5). In all cases, the animals were still socially connected olfactorily and auditorily, and the nesting conditions were the same as for the grouped animals.

### 2.3. Longitudinal Behavioral Assessment

All mice were evaluated following the same procedure in a pre- and post-longitudinal design at 13 months and again at 16 months. The corner test was used to evaluate the immediate neophobic response of the animals in a new standard home cage with a clear bed of wood save. Once the animal was placed in the new standard home cage, the following information was recorded immediately during the first 30 s: horizontal activity (number of visited corners) and vertical activity (number of rearings and latency of rearing). Behavioral assessment was performed from 9:00 a.m. to 1:00 p.m., counterbalanced and blinded to the experimenter.

### 2.4. Physical Status: Body Weight and Liver Index

Animals were weighed at the time of sacrifice, and their livers were dissected and stored at –80 °C until the protein extract was processed for biochemical analysis. The liver index or relative weight of the liver was calculated according to the following equation: Liver index = Weight of liver (g)/Bodyweight (g).

### 2.5. Histopathological Assessment

After euthanasia, a part of the liver was dissected, fixed with 10% formalin for 24 h, and embedded in paraffin for further histopathological analysis. Slices of 5 and 10 μm thickness were de-paraffinized and re-hydrated by several passes through ethanol at different concentrations.

#### 2.5.1. Morphological and Structural Analysis with H&E

Standard staining with hematoxylin and eosin (H&E) was performed, briefly, a 5-min incubation with Harris hematoxylin and a 2-min incubation with Eosin Y, with a dedifferentiation phase in between. Finally, the slices were manually mounted with DPX mounting medium after dehydration. Images were taken with a ×20 objective on a Nikon Eclipse 80i microscope, using a digital camera running on the control software ACT-1 (ver2.70).

#### 2.5.2. Congo Red Staining for Amyloidosis

Amyloidosis was assessed using an Amyloid Stain, Congo Red Kit (Sigma-Aldrich, Saint Louis, MO, USA). The staining procedure was performed according to the kit indications, with incubation in Mayer’s hematoxylin and then in Congo red solution. Finally, the slices were manually mounted with DPX mounting medium after dehydration. Images were taken with a ×20 objective on a Nikon Eclipse 80i microscope under normal light (bright field) and fluorescence light (fluorescent filter G-2A), using a digital camera running on the control software ACT-1 (ver2.70).

### 2.6. Biochemical Analysis

#### 2.6.1. HPA Axis: Glucocorticoids Measurements

Blood samples were collected from each animal during euthanasia. After centrifugation at 3500 rpm × 14 min, serum was preserved at −20 °C. Corticosterone levels (Ng/mL) were measured using a commercial kit (Corticosterone EIA Immunodiagnostic Systems Ltd., Boldon, UK). Absorbance was read at 450 nm using a Varioskan LUX ESW 1.00.38 (Thermo Fisher Scientific, Waltham, MA, USA).

#### 2.6.2. Hepatic Oxidative Stress

Antioxidant capacity was studied by evaluating the levels of total glutathione (GSH), the main non-enzymatic reducing agent of the organism, and glutathione peroxidase (GPx) and reductase (GR) enzymatic activity in liver homogenates, benefiting from the enzymatic recycling method previously described [40].

Total GSH was measured by absorbance at 412 nm and adapted to 96-well plates with slight modifications [41].

The enzymatic activity of glutathione reductase was assessed following the method described by Massey and Williams [42], with slight modifications. Monitoring the oxidation of NADPH spectrophotometrically was performed at 340 nm for 300 s.

The enzymatic activity of glutathione peroxidase was measured using a technique that was modified after a previous description [43,44]. The reaction was monitored spectrophotometrically by the decreasing absorbance at 340 nm for 300 s.

The results were expressed as milliunits (mU) of enzymatic activity per milligram (mg) of organ protein.

#### 2.6.3. Hepatic Inflammation

Liver homogenates were produced by lysing frozen liver samples in cold lysis buffer containing protease and phosphatase inhibitors (Sigma-Aldrich, Saint Louis, MO, USA). The protein content was quantified using the BCA Protein Assay Kit (Thermo Fisher Scientific, Waltham, MA, USA). TNF𝛼 and the cytokines IL-6 and IL-1𝛽 were quantified by sandwich ELISA using commercial kits (Biospes, Antibodies, Cambridge, UK). Absorbance was read at 450 nm using a Varioskan LUX ESW 1.00.38. (Thermo Fisher Scientific, Waltham, MA, USA).

### 2.7. Functional Correlates

The correlations between the functional variables, namely, behavioral phenotype, physical status, HPA axis, liver oxidative stress, and liver inflammation, were determined using Pearson’s test, considering the two intrinsic (genotype and sex) and extrinsic (isolation) factors. The data are shown as a heat map with correlation coefficients. The most important relationships with biological meaning are shown in scatter plots.

### 2.8. Statistics

Results are expressed as the mean ± SEM. GraphPad Prism 8.0 and R 4.1.2 software were used. Three-way and two-way analyses for multiple variables (ANOVA) and nested one-way (ANOVA) followed by Tukey’s post hoc test for multiple comparisons were used. Student’s t-test was used to assess the significance of differences between two independent groups. Functional correlations were analyzed using RStudio 1.4.1103 software. In all tests, statistical significance was set at *p* ≤ 0.05.

## 3. Results

### 3.1. Longitudinal Behavioral Assessment

**Figure 1:**
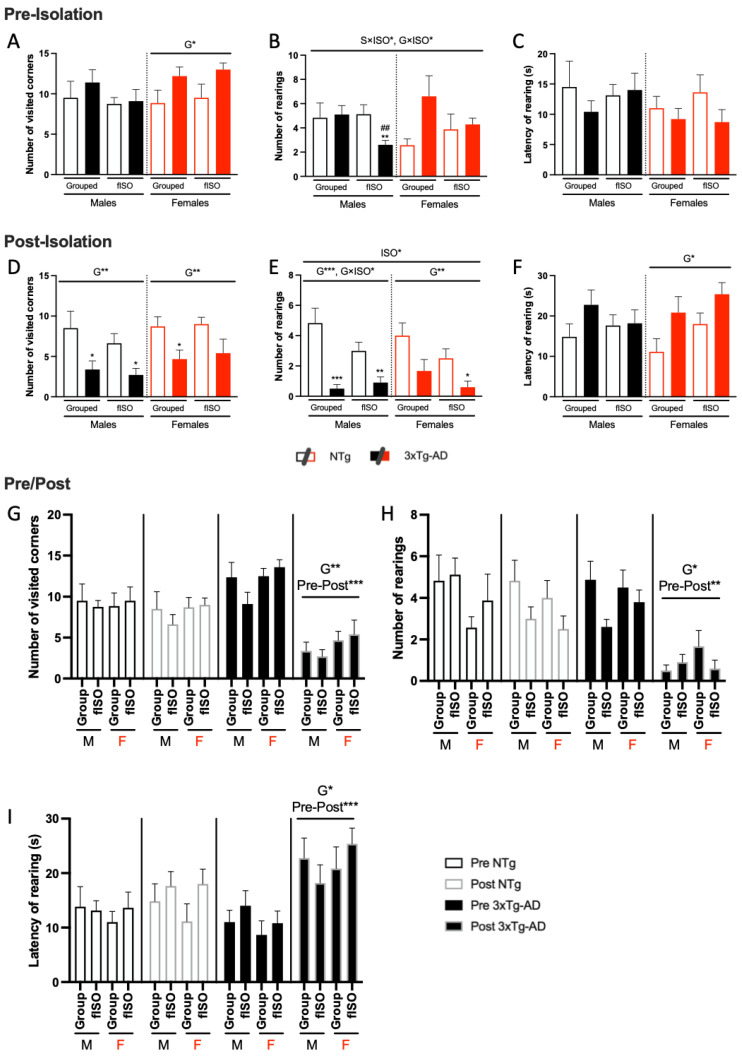
Pre- and post-isolation longitudinal behavioral assessment in the corner test for neophobia. Pre-isolation test: horizontal activity (**A**) and vertical activity (**B**,**C**). Post-isolation test: horizontal activity (**D**) and vertical activity (**E**,**F**). Comparison between pre- and post-test in horizontal activity (**G**) and vertical activity (**H**,**I**). N = 7–10 mice per group, mean ± SEM. Genotype: G*, *p* ≤ 0.05; G**, *p* ≤ 0.01; G***, *p* ≤ 0.001. Forced isolation: ISO*, *p* ≤ 0.05. Interaction sex and isolation: S × ISO* *p* ≤ 0.05. Interaction genotype and isolation: G × ISO* *p* ≤ 0.05. Pre- and post-differences: Pre-Post**, *p* ≤ 0.01; Pre-Post***, *p* ≤ 0.001. Differences between two counterparts, genotype: * *p* ≤ 0.05, ** *p* ≤ 0.01, *** *p* ≤ 0.001 vs. NTg counterpart; isolation: ## *p* ≤ 0.01 vs. grouped counterpart.

The pre- and post-isolation assessment of the horizontal activity (number of visited corners) and vertical activity (number of rearings and latency of rearing) of the neophobia response were longitudinally assessed. At the age of 13 months, genotype differences were already observed among the females (Figure 1A; F (1, 28) = 6.285, *p* = 0.0183), with an increased number of visited corners in 3xTg-AD females as compared to their NTg counterparts. At 16 months, genotype effects were found for both sexes in horizontal and vertical counts (Figure 1D–F; all F’s (1, 22) ≥ 6.784, *p* = 0.0152). A genotype × isolation interaction effect (Figure 1E; F (1, 28) = 4.241, *p* = 0.0489) was present in the number of rearings in males. Further statistical analysis revealed that these genotype effects were attributable to a strong reduction in the number of visited corners and rearings in all 3xTg-AD males. In contrast, only grouped females showed a statistically significant reduction in horizontal activity, while isolated 3xTg-AD females showed this reduction in vertical activity. The isolation effect was shown by the number of rearings (Figure 1E; F (1, 50) = 5.1, *p* = 0.0283), with fISO groups exhibiting decreased vertical activity. (Figure 1G–I; all F’s (3, 12) ≥ 9.008, *p* = 0.002) showed pre- and post-differences that were clearly manifested in the 3xTg-AD animals. No sex differences were found (all F’s (3, 12) ≥ 9.008, *p* = 0.0021).

### 3.2. Physical Status: Body Weight and Liver Index

**Figure 2:**
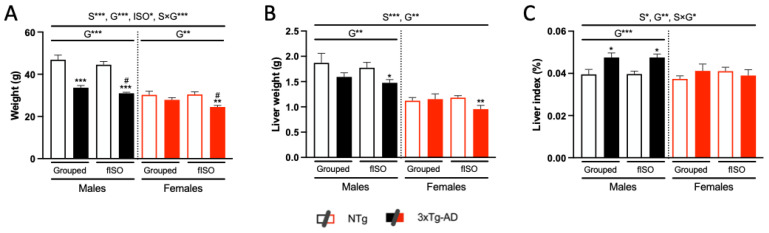
Physical status: Body (**A**) and liver (**B**) weights and liver index (**C**). N = 7–10 mice per group, mean ± SEM. Sex: S*, *p* ≤ 0.05; S***, *p* ≤ 0.001. Genotype: G**, *p* ≤ 0.01; G***, *p* ≤ 0.001. Forced isolation: ISO*, *p* ≤ 0.05. Interaction sex and genotype: S × G* *p* ≤ 0.05; S × G*** *p* ≤ 0.001. Differences between two counterparts, genotype: * *p* ≤ 0.05, ** *p* ≤ 0.01, *** *p* ≤ 0.001 vs. NTg counterpart; isolation: # *p* ≤ 0.05 vs. grouped counterpart.

Sex and genotype effects were found in all the variables studied (Figure 2; S, all F’s (1, 58) ≥ 5.309, *p* = 0.0248; G, all F’s (1, 58) ≥ 7.140, *p* = 0.0098), and interaction between these factors occurred in the body weight of the animal and the liver index (Figure 2A,C; all F’s (1, 58) = 4.721, *p* = 0.0339). Body weight measurements (Figure 1A) showed sexual dimorphism. The genotype effect, with a higher body weight recorded in NTg mice, was mainly due to males, whereas genotype differences in females were due to reduced weight in isolated animals. Similar differences were observed in the liver weight (Figure 2B). Thus, genotype differences in liver weight were enhanced, reaching statistical significance, in both male and female isolated animals compared to their NTg isolated counterparts. The relative weight of the livers revealed hepatomegaly in the 3xTg-AD mice, and this effect was primarily due to male sex (Figure 2C), in addition to sexual dimorphism. The effect of isolation was found in body weight, which was due to the reduction observed in the 3xTg-AD mice.

### 3.3. Histopathological Assessment: H&E and Congo Red Staining

**Figure 3:**
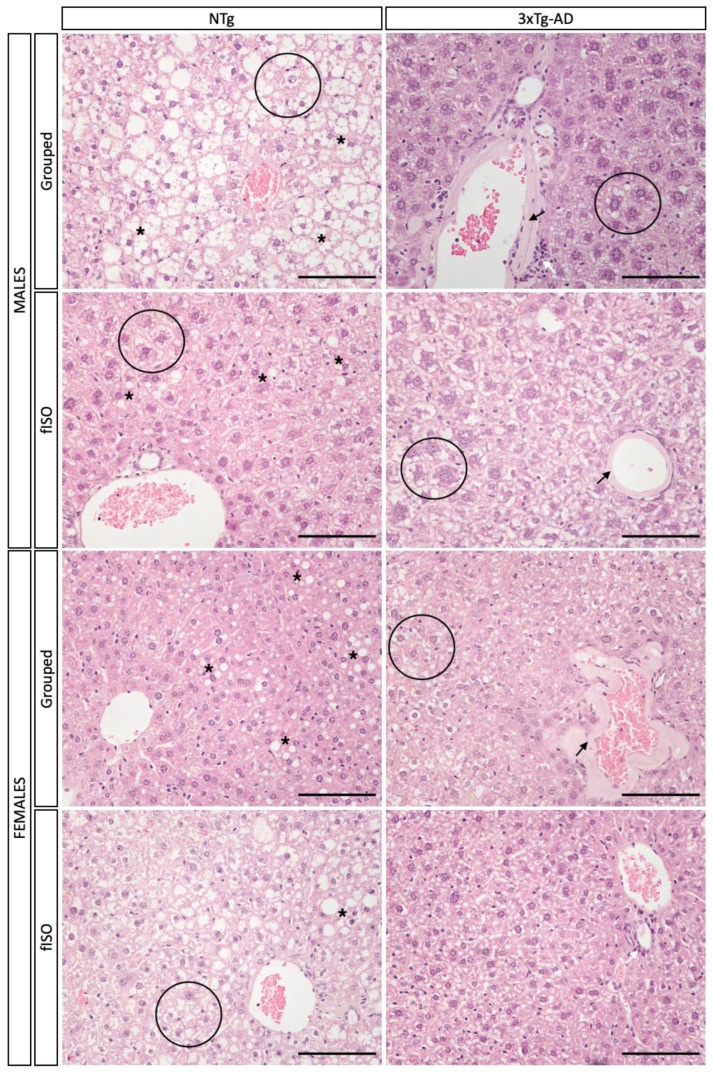
H&E staining of liver tissue. Morphological and structural comparison of liver tissue in NTg and 3xTg-AD males and females grouped and those submitted to forced isolation. H&E stained. Symbols indicate the morphological features as follows: *—steatosis; black arrow—amyloidosis; white arrow—inflammatory infiltration; black circle—ballooning. The images were taken with ×20 objective lens.

**Figure 4:**
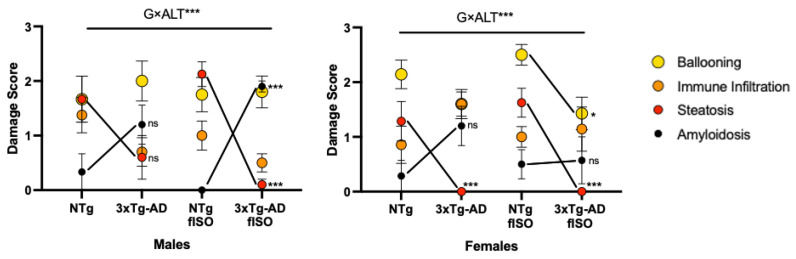
Histopathological damage score of liver tissue. Comparison between NTg and 3xTg-AD males and females and those submitted to forced isolation. The score was determined according to the severity presented in four morphological alterations: ballooning, immune infiltration, steatosis, and amyloidosis, each receiving a score between 0 and 4 for each animal. N = 7–10 mice per group, mean ± SEM. Genotype (G) and morphological alterations (ALT) interaction effect: G × ALT*** *p* ≤ 0.001. Differences between two counterparts: * *p* ≤ 0.05, *** *p* ≤ 0.001 vs. NTg counterparts.

Histopathological sections were prepared and stained for microscopic analysis to determine morphological and physiological alterations in the liver. Two genotype-dependent morphological alterations were observed (Figure 3 and Figure 4; all F’s (3, 56) = 3.840, *p* = 0.0143). In NTg mice, the main alteration observed was accumulation of lipids in the form of fat droplets, indicating a state of steatosis and possibly an early stage of non-alcoholic fatty liver disease (NAFLD). Steatosis was present in all NTg groups to varying degrees, tending to affect more males than females. In contrast, in 3xTg-AD mice, excessive thickening of the blood vessel parenchyma, losing its typical circular structure due to amyloidosis, tended to be the main alteration, reaching statistical significance in isolated males. Isolation in males caused the change in these alterations by genotype to be more pronounced, while in females, the only effect was the attenuation of amyloidosis in 3xTg-AD females. Infiltration of immune cells was observed in all groups, suggesting a possible inflammatory state in the liver. Additionally, cell damage in the form of ‘‘ballooning’’ was observed in all animals regardless of sex and genotype, although an isolation effect was found in 3xTg-AD females, reducing this damage.

**Figure 5:**
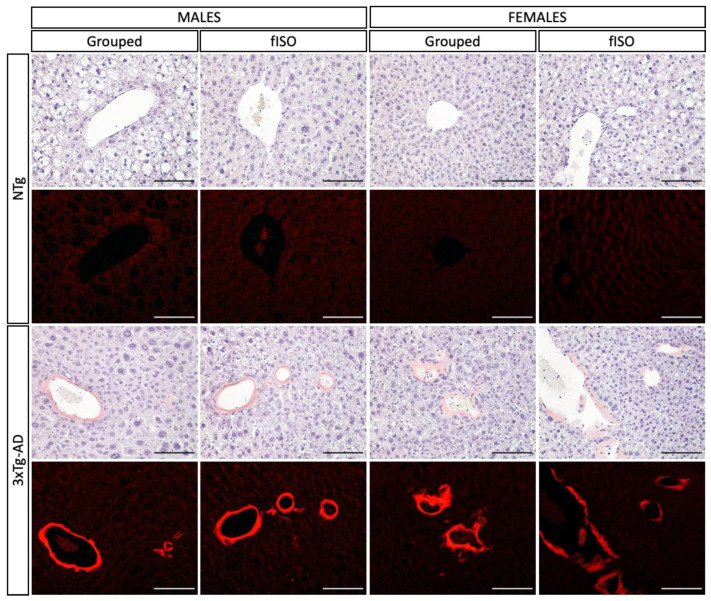
Congo red staining of liver tissue. Differences in vascular parenchyma of liver tissue in NTg and 3xTg-AD males and females grouped and those submitted to forced isolation. Congo red stained. The images were taken with ×20 objective lens; magnification bar = 100 μm.

Congo red staining was performed to detect amyloidosis in the liver. As shown in Figure 5, in the bright field images, NTg animals did not show stained areas, whereas 3xTg-AD animals showed parenchyma of the blood vessels with the characteristic reddish tone of this staining. In addition, when the sections were viewed under fluorescent light, the areas marked by the staining showed intense red fluorescence.

### 3.4. Biochemical Status of the Liver–Brain and HPA Axis

**Figure 6:**
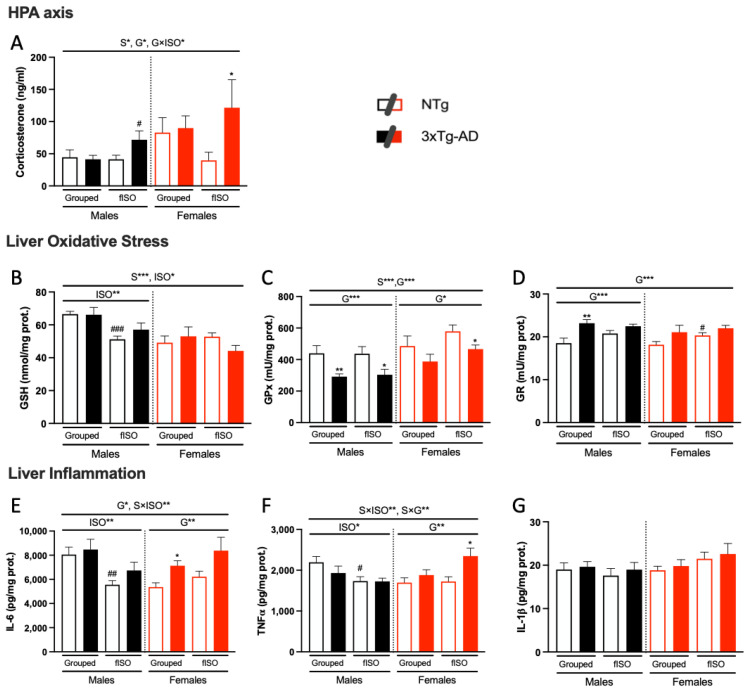
Biochemical analysis of the liver–brain and HPA axis. Corticosterone levels in serum (**A**). Total levels of GSH in the liver (**B**). Enzymatic activity of GR and GPx (**C**,**D**, respectively). Pro-inflammatory cytokines levels of IL-6 (**E**), TNF𝛼 (**F**), and IL-1𝛽 (**G**). N= 7–10 mice per group, mean ± SEM. Sex: S*, *p* ≤ 0.05; S***, *p* ≤ 0.001. Genotype: G*, *p* ≤ 0.05; G**, *p* ≤ 0.01; G***, *p* ≤ 0.001. Forced isolation: ISO*, *p* ≤ 0.05; ISO**, *p* ≤ 0.01. Interaction effects: sex and genotype: S × G**, *p* ≤ 0.01; sex and isolation: S × ISO**, *p* ≤ 0.01; sex and genotype: S × G**, *p* ≤ 0.01. Differences between two counterparts, genotype: * *p* ≤ 0.05, ** *p* ≤ 0.01 vs. NTg counterpart; isolation: # *p* ≤ 0.05, ## *p* ≤ 0.01, ### *p* ≤ 0.001 vs. grouped counterpart. G × ISO*, *p* ≤ 0.05.

The corticosterone levels of NTg and 3xTg-AD mice living in standard housing conditions did not differ. However, genotype differences emerged in the groups subjected to isolation, resulting in an interaction between genotype and isolation, with increased levels in both male and female 3xTg-AD mice compared to their NTg counterparts (Figure 6A; F (1, 58) = 4.352, *p* = 0.0414). Sexual dimorphism, with higher levels in females than in males, was also observed (Figure 6A; F (1, 58) = 5.525, *p* = 0.0222).

Liver oxidative stress was assessed by measuring GSH levels and GR and GPx enzymatic activity. Sexual dimorphism was observed in GSH levels and GPx activity (Figure 6B,C; all F’s (1, 58) ≥ 12.4, *p* = 0.0009), with females having lower levels of GSH and higher GPx activity. An isolation effect was also observed in the GSH levels (Figure 6B; F(1, 57) = 6.178, *p* = 0.0159), which were reduced in the isolated mice. Genotype differences were found for both GPx and GR enzymatic activities (Figure 6C,D; all F’s (1, 28) = 5.056, *p* = 0.0326) were more marked in GPx activity. The 3xTg-AD animals had reduced GPx activity and increased GR activity compared to NTg animals. Therefore, the AD genotype affects liver metabolism in terms of oxidative stress, altering the functioning of antioxidant defenses.

Liver inflammation, on the other hand, was assessed through the levels of IL-6, TNF𝛼, and IL-1𝛽. Genotype and isolation had differential effects according to sex (Figure 6E,F; S × ISO F (1, 56) = 8.407, *p* = 0.0053; S × G F (1, 56) = 7.326, *p* = 0.009). Genotype had an effect on females (Figure 6E,F; all F’s (1, 55) ≥ 6.465, *p* = 0.0138), resulting in higher levels of IL-6 and TNF𝛼 in 3xTg-AD animals. Isolation had an effect on males (Figure 6E,F; all F’s (1, 29) ≥ 5.927, *p* = 0.0213), showing lower levels of IL-6 and TNF𝛼 in isolated males compared to grouped males. IL-1𝛽 levels were not affected. In males, the levels were very similar in all groups, whereas in females, there was a slight tendency to increase, although the difference was not statistically significant.

Additionally, due to the death of their cage mates, a group of transgenic males was isolated (Males 3xTg-AD nISO) and added to the study to determine the effect of two different levels of isolation (Table 1). The results of the variables analyzed for this group followed the trend observed with the fISO. Only forced isolation achieved statistical significance in comparison to its grouped counterpart in body weight (Table 1; t (18) = 2.201, *p* = 0.041), consisting in a reduction.

### 3.5. Meaningful Functional Correlates between Behavioral, Physical Metrics, Liver Oxidative Stress, and Inflammation Variables

**Figure 7:**
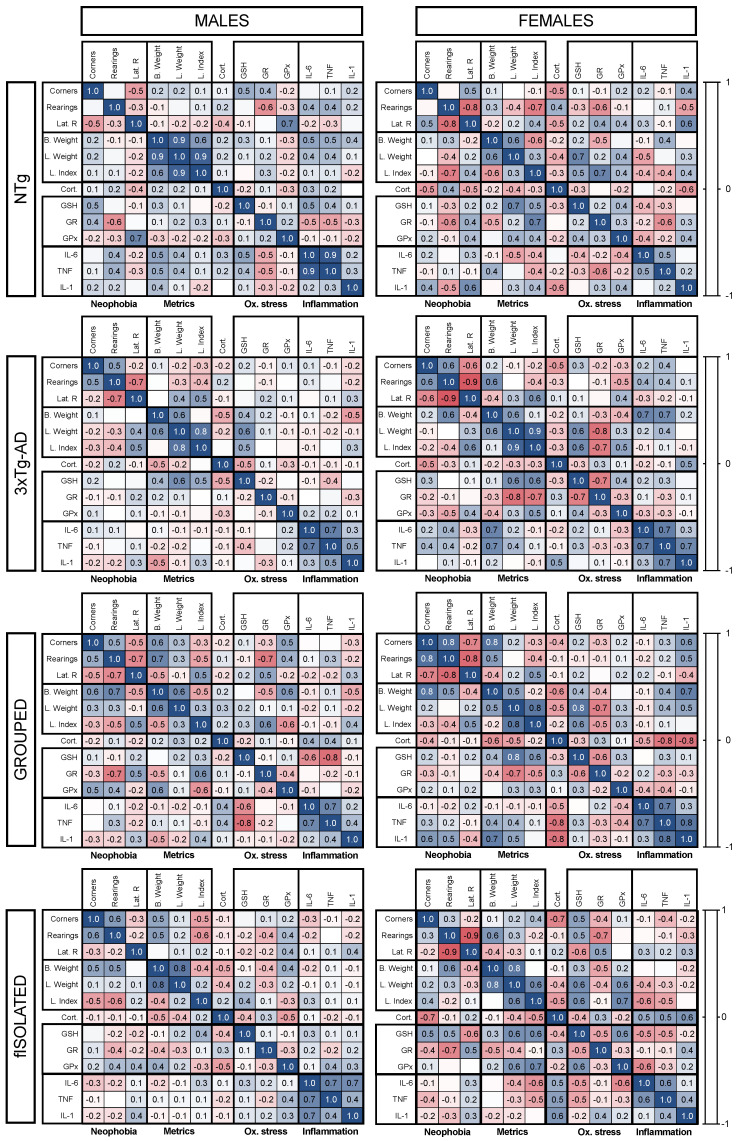
Heat map of correlations between the studied variables. Neophobia: number of visited corners, number of rearings, and latency of rearing; physical metrics: body weight, liver weight, and liver index; corticosterone; oxidative stress: GSH levels, GR activity, and GPx activity; inflammation: IL-6, TNF𝛼, and IL-1𝛽 levels. The color scale shows the correlation intensity between 1 and −1, with positive correlations in blue and negative correlations in red. The statistical analysis was based on Pearson’s test.

**Figure 8:**
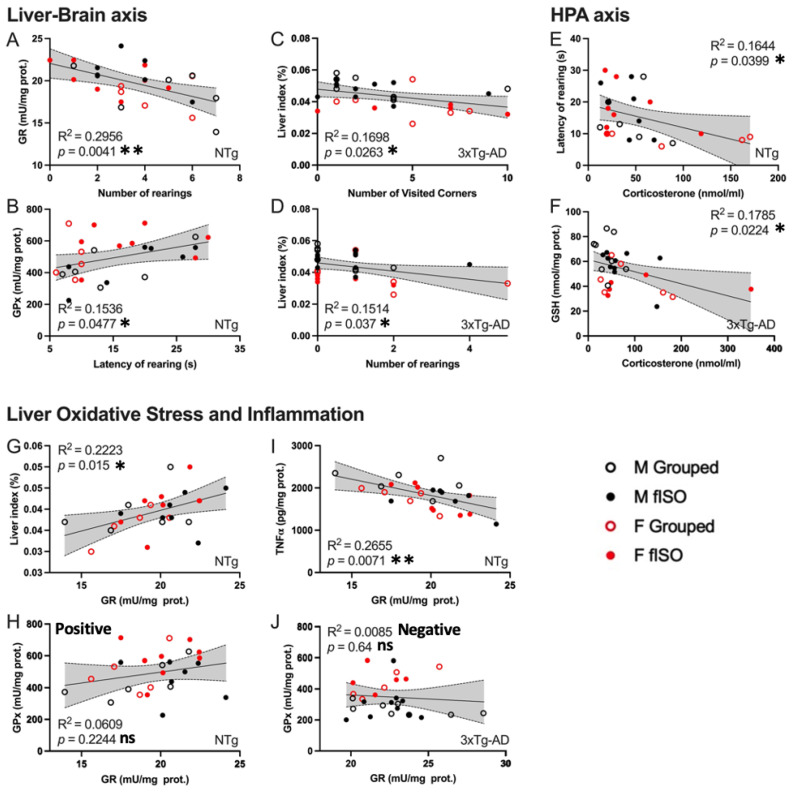
Scatter plot representing the most biologically relevant correlations: GR activity and number of rearings by NTg animals (**A**); GPx activity and latency of rearings by NTg animals (**B**); liver index and number of corners visited by 3xTg-AD animals (**C**); liver index and number of rearings by 3xTg-AD animals (**D**); latency of rearing and corticosterone levels in NTg animals (**E**); GSH levels and corticosterone leves in 3xTg-AD animals (**F**); liver index and GR activity in NTg animals (**G**); TNF𝛼 levels and GR activity in NTg animals (**H**); GPx activity and GR activity in NTg animals (**I**); GPx activity and GR activity in 3xTg-AD animals (**J**). N = 6–10 mice per group, each linear regression includes the correlation index (R^2^) and the level of significance of the correlation (*p*), * *p* ≤ 0.05, ** *p* ≤ 0.01.

Statistical correlation tests were performed between all variables, separated by the different factors considered in the study, to determine the possible relationships (Figure 7). Next, relationships were established between the functional variables with biological significance and how they behaved according to the genotype (Figure 8).

These correlations varied according to sex, genotype, and isolation (Figure 7). In all cases, it can be seen how relationships changed in intensity and how some changed from positive to negative, or vice versa. Females established more and stronger relationships between the variables than males. This was more pronounced in the 3xTg-AD animals, where males presented 26.92% of null relationships (correlation equal to 0) and females presented 7.69%. The genotype effect observed differed depending on sex. Thus, in males, the range of null relations increased from 10.26% in NTg mice to 20.51% in 3xTg-AD mice, while in females the opposite occurred, going from 12.82% in NTg to 7.69% in 3xTg-AD females. Regarding for the effect of isolation on the relationships between variables, no remarkable changes were observed, although sexual dimorphism was preserved. In this case, if strong positive and negative relationships (with coefficients between 0.5 and 1) are considered, isolated males accounted for 7.69% and 5.13%, respectively, while isolated females accounted for 19.23% and 15.38%, respectively.

In view of this and our focus on the possible relationships between behavior, physical status, oxidative stress, and inflammation in physiological and AD aging, the most relevant correlations were further analyzed. Starting with the relationship between the brain and liver, NTg animals with physiological aging present a connection between the variables studied with the corner test and oxidative stress variables. Specifically, the number of rearings and rearing latency were related to the enzymatic activity of antioxidant enzymes (Figure 8A,B). In the 3xTg-AD animals, in the other hand, a relationship was established using the liver index (Figure 8C,D). Regarding the HPA axis, in NTg animals, it was related to rearing latency but not to oxidative stress or inflammation. In 3xTg-AD animals, the opposite occurred, relating to GSH levels but not to behavior variables (Figure 8E,F). If we focused on oxidative stress and inflammation in the liver, it was observed in NTg animals that the liver index was related to GR activity (Figure 8G), which was also related to TNFα levels (Figure 8I). Finally, it should be noted that a mismatch between the activity of both anti-inflammatory enzymes was observed. In NTg animals, both enzymes that are part of GSH antioxidant cycle had a positive relationship (Figure 8H). However, in 3xTg-AD animals this relationship was lost, indicating that the activity of both enzymes was unbalanced in the 3xTg-AD genotype (Figure 8J).

## 4. Discussion

AD is one of the most important neurodegenerative diseases, with extensive literature on brain changes, in contrast to research on the effects or involvement of peripheral organs. The liver, the main organ responsible for metabolism, is one of the most susceptible organs to AD [45]. Previous studies by our group have shown peripheral alterations at prodromal (4 months of age) and early stages of disease (6 months of age) in oxidative stress in 3xTg-AD mice, suggesting accelerated aging. However, these genotype differences were lost at very late ages (18 months), when physiological AD neuropathological aging seems to converge [19,20,27,46]. This study assessed the histopathological profile of mice at 16 months of age, which is a very advanced stage of the disease. Furthermore, oxidative stress status and inflammation in the liver of 3xTg-AD mice were assessed to determine the relationship between the AD genotype and this organ. The impact of sex and isolation as intrinsic (biological) and extrinsic (environmental) factors, respectively, have also been studied. In addition, old male C57BL/6 mice have been used as animal models of aging [47].

### 4.1. Hepatic Pathology Changes according to Genotype

The physical status and histopathology of the liver indicated that this organ’s state was disturbed in both 3xTg-AD and NTg mice, but in a different manner. NTg mice, the gold-standard C57BL/6 mice, as the genetic background of our 3xTg-AD mice colony, showed the highest body and liver weights, indicating an obese phenotype. This genetic background has been widely used to develop mouse models predisposed to obesity and other liver disorders [48,49], which is consistent with our results considering the old age of the animals. Massive accumulation of lipids was present in their livers, an event commonly seen in various liver diseases that have been associated with aging and obesity, such as NAFLD. This disease is characterized by lipid deposition, leading to steatosis, which causes oxidative stress and inflammation, and ultimately leads to NASH [50,51]. Hepatic steatosis increases liver size and weight, which is commonly referred to as hepatomegaly [52].

These effects were expected to be more marked in the 3xTg-AD animals, where hepatomegaly was more marked. Surprisingly, the outcome was the opposite, since 3xTg-AD animals presented lower grades of steatosis, and instead, they presented with amyloidosis, a group of diseases caused by the extracellular deposition of insoluble amyloid fibrils. Congo red has been shown to be a reliable stain for the detection of amyloid plaques [53] and diagnosis of amyloidosis [53,54]. In this study, most amyloid accumulation was observed in the portal fields of 3xTg-AD mice in the form of vascular and interstitial deposits. From the clinical manifestation of amyloidosis, it is known that it can develop in a systemic way affecting several organs, such as the liver, leading to hepatocellular atrophy and progressive organ failure [55,56]. The liver could be one of the organs most affected, since one of its primary functions is to detoxify noxious compounds that are toxic to the organism [57]. Moreover, hepatocytes are involved in the process promoting Aβ clearance by degradation or by excretion with the bile [58]. The accumulation observed in the portal fields denotes a failure in this clearance of Aβ by the liver. The 3xTg-AD mouse model carries two transgenes for β-amyloid pathology [22], and there is evidence to suggest that Aβ is not only restricted to the brain but can also develop in other peripheral organs, such as the liver. In fact, Aβ from the brain can be transported to these peripheral organs via the brain–blood barrier (BBB) or the blood–CFS barrier, which we demonstrated to be disrupted in 3xTg-AD mice at this age [15,59]. 3xTg-AD males also had hepatomegaly, as shown by their liver index, which is considered a manifestation of amyloidosis in this mouse model [60]. The present results establish a point of connection between both organs in this mouse model and for both sexes, providing further evidence that reinforce the relevance of the liver–brain axis in AD.

Hepatocellular damage, characterized by swelling of the hepatocytes and clearing of the cytoplasm is called ‘’ballooning’’, and occurs when the liver suffers severe damage and the cells begin to degenerate [61]. Interestingly, ballooning was observed in all animals and therefore considered not genotype-dependent. This is an alteration associated with NASH [62] and could explain the affectation of NTg animals.

### 4.2. Hepatic Oxi-Inflammation Metabolic Disruption

GSH is one of the most important antioxidant peptides in the liver to manage oxidative stress [63,64], and the GSSG/GSH ratio can be used as a rutinary biomarker of oxidative stress in hepatocytes [65]. In the present study, the possible increase in oxidative stress was evaluated by the state of the cycle of GSH. Under normal conditions, this peptide is almost completely reduced; however, under stress conditions, it is oxidized by the action of GPx and reduced by the action of GR, forming a mechanism that aims to reduce reactive species within the cell [66]. GSH also promotes the expression of antioxidant genes [67]. Although sex differences and isolation effects were observed in GSH levels, the lack of genotype changes suggests the same level of protection from oxidative stress in both NTg and 3xTg-AD animals. Interestingly, GPx activity, which is responsible for the detoxification of reactive species due to the oxidation of GSH [68], was reduced in 3xTg-AD animals, indicating a loss in antioxidant capacity. Our group determined peripheral alterations in the oxidative stress status of male and female 3xTg-AD mice occurring at the prodromal (4 months of age) and early stages of the disease (6 and 9 months of age). These alterations consisted of a reduction in GSH levels as well as a reduction in GPx and GR enzyme activity in the liver, spleen, and kidney. In addition, the levels of xanthine oxidase also increased [19,20,27]. This is consistent with the results of this study, where the reduction in GR activity was preserved at 16 months of age at a much more advanced stage of the disease. However, the differences in GSH levels were lost, and GPx enzymatic activity increased rather than decreased. This indicated that the liver continued to exhibit persistent oxidative stress. All differences in oxidative stress were lost by the age of 18 months, suggesting that at this age NTg animals reached the level of oxidative stress in 3xTg-AD animals [46].

Another important aspect to consider is inflammation, as it has been widely reported to have a close relationship with oxidative stress in this organ [15,69]. Indeed, we found that the levels of these cytokines were increased in 3xTg-AD females, indicating that this could be related to the levels of corticosterone and oxidative stress in the liver.

### 4.3. HPA Axis Disruption, Oxidative Stress, and Inflammation in the Liver

The HPA axis is responsible for psychological stress responses; alterations in this axis may be reflected in cognitive impairment, oxidative stress, and inflammation. Anxiety and psychological factors are among the most relevant psychological factors in patients with AD. Psychological stress also disrupts the HPA axis [70,71]. These psychological effects must be considered when evaluating the relationship between AD and liver function. One advantage of using animal models is that they can reproduce an important number of characteristics observed in humans [38,72]. In particular, the 3xTg-AD animals exhibited marked neophobia at 16 months of age, and their performance indicated worsening of long-term memory in such an experimental scenario, considering that the animals were already tested at 13 months. Moreover, anxiety, depression, and stress-like behavior are also present [24,72].

3xTg-AD female mice at 4 months of age at the initial stages of AD already show HPA axis hyperactivity and overproduction of corticosterone [11,73]. This study also found this difference in 3xTg-AD females, with a tendency toward higher corticosterone levels. Moreover, this is consistent with previous findings from our group, where old females presented higher levels of corticosterone than males, suggesting that these sex differences in corticosterone levels may be triggered by estrogens [23]. Corticosterone also influences oxidative stress and inflammation. Chronically increased corticosterone levels alter lipid metabolism and oxidative phosphorylation in liver mitochondria, leading to oxidative stress [74]. Isolated perfused livers from rats exposed to psychological stress showed an increase in tumor necrosis factor (TNF𝛼) and interleukin-6 (IL-6) [75]. This highlights the relationship between corticosterone and oxidative stress and inflammation, which would agree with the results obtained from the biochemical analysis of 3xTg-AD females.

### 4.4. Meaningful Functional Correlates between Behavioral, Physical Metrics, Liver Oxidative Stress, and Inflammation Variables

The statistical correlations shown in the results help us understand how the variables behave with each other and can provide information on the possible relationship between AD genotype and alterations observed in the liver.

It can be seen that there is a relationship between behavior and the physiological processes that are happening in the liver. The involvement of cognitive processes in the physiology of peripheral organs is beginning to be studied. In humans, the connection between the central nervous system and periphery through fear has already been determined [76,77]. In this study, we observed that neophobia was directly related to the activity of antioxidant enzymes. In addition, 3xTg-AD animals showed high neophobia, which was correlated with the liver index, establishing a link between behavior and hepatomegaly, possibly through amyloidosis in the liver. On the other hand, another axis of connection could also be established through corticosterone. In 3xTg-AD animals, a negative correlation was observed between corticosterone and GSH levels, indicating a relationship between corticosterone levels and the oxidative status of the liver. In rat models with a chronic glucocorticoid-enriched endogenous environment, it has already been shown that oxidative stress in the liver is increased by a reduction in GSH levels [78]. Furthermore, this is consistent with what we observed for the relationship between GR and GPx enzymatic activity, since in NTg animals, the activity of both enzymes is timed for the cycle to work, but in 3xTg-AD animals this relationship is lost. This imbalance in the activity of these two enzymes caused by the AD genotype indicates that the reduction in reactive species is not efficient and therefore increases the oxidative stress generated in the liver. This imbalance in the activity of these two enzymes caused by the AD genotype indicates that the reduction in reactive species is not efficient and therefore increases the oxidative stress generated in the liver. This has already been observed in the brains of mice following severe ethanol administration. This causes the overproduction of reactive oxygen species that impairs the antioxidant system by reducing GPx activity and increasing GR activity, which is considered a compensatory mechanism to preserve the cell oxidative stress status [79]. Finally, excess accumulation of lipids in the liver, known as steatosis, impairs antioxidant defenses that eventually cause liver damage due to oxidative stress and simultaneously activate the entire pro-inflammatory machinery. This damage leads to the development of chronic liver diseases, particularly NAFLD [80,81]. This is consistent with our histopathological and biochemical results for NTg animals and becomes evident with the correlations between the liver index, enzymatic activity of antioxidant enzymes, and levels of pro-inflammatory factors. Despite not presenting with this liver disease, 3xTg-AD animals were found to be affected by a group of diseases known as amyloidosis. Based on these results, oxidative stress and inflammation were found to be exacerbated.

The paradigm comes when we focus on the effect of social isolation as an environmental factor in the model. Many patients with AD face situations of social isolation that imply major psychological stress in addition to the intrinsic stress generated by the disease [82]. In our experimental scenario, the isolated 3xTg-AD mice showed a similar pattern of neophobia as the grouped mice. This contrasts with the results obtained in the group’s previous studies, where isolated mice exhibited considerable changes in the cognitive tests performed, indicating that they suffered hyperactivity, bizarre behaviors, anxiogenic patterns, and stress-coping responses that shifted to flight behavior [33]. Histopathological and biochemical analysis of the liver showed a main effect increasing the genotype difference. The different isolation scenarios were compared, and no differences were found between naturalistic and forced isolation in the study variables, except in body weight. Weight reduction was observed in forced-isolated animals, which was not as marked in naturally isolated animals.

## 5. Conclusions

This longitudinal study confirms the existence of the liver–brain axis alterations in Alzheimer’s disease and identifies hepatic oxi-inflammation and neophobia as potential integrative system targets that are under the modulation of intrinsic (genotype and sex) and extrinsic (social conditions) factors. In particular:

Hepatic dysfunction was determined at the organ, tissue, and cellular levels in 16-month-old animals. The hallmarks of 3xTg-AD liver dysfunction included hepatomegaly, acute amyloidosis, and ballooning. In addition, increased oxidative stress and inflammation were shown as the dysregulation of antioxidant enzymes and increased levels of pro-inflammatory cytokines, with a sexual dimorphism. In contrast, liver steatosis was present in their male and female C57BL/6 wild-type counterparts and, in the case of males, was co-morbid with obesity.

Dysregulation of the HPA axis may also be involved in liver oxidative stress and inflammation, as shown by the higher corticosterone levels in 3xTg-AD females, which correlated with GSH levels.

Neophobia has previously been shown to exert a clear psychological effect by increasing hyperactivity, anxiogenic patterns, and bizarre/flight behavior. In this study, neophobia correlated with oxidative stress variables in the liver.

In forced isolated animals, the genotype differences on hepatic histopathology (liver damage score) and oxi-inflammation mechanisms (reduced GSH levels and increased IL-6 and TNF𝛼) were enhanced or appear for the first time.

The liver–brain axis is an emerging research area in AD that demands further efforts to unveil the interplay of the pathways involved the systemic component of this disease.

## Figures and Tables

**Table 1:** Effect of different isolation scenarios, naturalistic (nISO) and forced (fISO), in 3xTg-AD males with AD-pathological aging. Number of visited corners; number of rearings; latency of rearing; body weight; liver weight; liver index; GSH levels; GR activity; GPx activity; IL-6 levels; TNF𝛼 levels and IL-1𝛽 levels. N = 5–10 mice per group, mean ± SEM. Isolation: # *p* ≤ 0.05.

	3xTg-AD, Male Grouped (*n* = 8) Mean ± SEM	3xTg-AD, Male ISO (*n* = 15) Mean ± SEM	3xTg-AD, Male fISO (*n* = 10) Mean ± SEM	3xTg-AD, Male nISO (*n* = 5) Mean ± SEM
Visited corners	3.38 ± 1.07	3.00 ± 0.59	2.70 ± 0.82	3.60 ± 0.75
Rearings	0.50 ± 0.27	1.13 ± 0.34	0.90 ± 0.38	1.60 ± 0.68
Lat. Rearing (s)	22.75 ± 3.70	16.93 ± 2.83	18.20 ± 3.31	14.40 ± 5.70
Body weight (g)	33.60 ± 1.08	31.27 ± 0.49 #	30.97 ± 0.51 #	31.88 ± 1.12
Liver weight (g)	1.59 ± 0.08	1.43 ± 0.05	1.48 ± 0.07	1.33 ± 0.09
Liver index (%)	0.05 ± 0.002	0.05 ± 0.001	0.05 ± 0.002	0.4 ± 0.002
GSH (nmol/mg prot.)	66.13 ± 23.19	55.19 ± 3.46	57.12 ± 4.06	51.34 ± 6.76
GR (mU/mg prot.)	23.19 ± 0.86	22.85 ± 0.51	22.49 ± 0.46	23.57 ± 1.24
GPx (mU/mg prot.)	291.46 ± 17.49	300.90 ± 26.93	303.58 ± 35.02	295.55 ± 45.67
IL-6 (pg/ mg prot.)	8742.12 ± 847.56	7125.19 ± 696.54	6735.39 ± 689.15	7904.79 ± 1652.04
TNF𝛼 (pg/ mg prot.)	1930.90 ± 169.66	1769.08 ± 101.78	1727.44 ± 79.16	1852.35 ± 279.25
IL-1β (pg/ mg prot.)	19.63 ± 1.20	18.92 ± 1.22	18.98 ± 1.65	18.81 ± 1.81

## Data Availability

The data presented in this study are available on request to the corresponding author.
